# Extraction and Optimization of Lycopene From Selected Fruits and Their Assessment as an Ultraviolet Ray Protectant for *Escherichia coli*

**DOI:** 10.1002/fsn3.70090

**Published:** 2025-04-18

**Authors:** Anil Kumar, Niharika Bhawsar, Savita Manekar, Bharat Pendram, Pritibala Pal, Daoud Ali, Saud Alarifi, Shivraj Gangadhar Wanale, Suchi Singh, Shailaja Katare, G. M. Srivastava, Parwiz Niazi, Sakshi Pareek, Virendra Kumar Yadav

**Affiliations:** ^1^ Department of Botany and Zoology Govt. Tilak P G College Katni Madhya Pradesh India; ^2^ Department of Zoology Govt. Narmada College Narmadapuram Madhya Pradesh India; ^3^ Department of Zoology University of Allahabad Prayagraj Uttar Pradesh India; ^4^ Department of Microbiology J H Govt. P G College Betul Madhya Pradesh India; ^5^ Department of Zoology College of Science, King Saud University Riyadh Saudi Arabia; ^6^ Department of Chemistry Netaji Subhashchandra Bose Art's, Commerce and Science College Nanded Maharashtra India; ^7^ Department of Biology Faculty of Education, Kandahar University Kandahar Afghanistan; ^8^ Department of Chemistry Wageningen University & Research Wageningen; ^9^ Marwadi University Research Center, Department of Microbiology Faculty of Sciences, Marwadi University Rajkot Gujarat India

**Keywords:** carotenoids, inhibition, lycopene film, petroleum ether, UV‐ray blocking

## Abstract

Lycopene is known for protecting the skin from harmful UV rays, which is why it is widely used in cosmetics. In the present investigation, fruits and vegetables like *Capsicum annuum* (*C.a.*), *Capsicum frutescens* (*C.f.*), *Carica papaya* (*C. p.*), *Citrullus lanatus* (*C. l.*), and *Solanum lycopersicum* (*S. l.*) were used for the extraction and isolation of lycopene. The lycopene was isolated in acetone to solubilize hydrophilic and hydrophobic molecules and hydrolyzed into 5% sodium sulfate in a protic solvent mechanism. The measurement of lycopene content was done by UV–Vis spectrophotometer at 503 nm (*λ*), and the absence peak was in the range of 440–530 nm for the extracted lycopene. Furthermore, all the Petri plates were coated with a film of lycopene extracted at different concentrations on the bottom surface of the lid of the Petri plate. The concentrations of lycopene present in the samples were in the following ascending order from lowest to highest concentration: (*C.a.*) < (*C.f.*) < (*C. p.*) < (*C. l.*) < (*S. l.*). The pH value of the *S. lycopersicum* (*S. l.*) sample was found to be 6.2, the maximum amount of lycopene was found to be 3.36024 μg/mL, and the pH value of the *C. annuum* (*C.a.*) sample was found to be 4.5 and the minimum amount. Lycopene was found to be 0.31824 μg/mL. The R‐square of the obtained pH value was increased with the adequate amount of lycopene of good quality; the correlation variables between the pH value and lycopene percentage remained the same using the equation *y* = 56.60×−250.5, and their *R* ^2^ value was 1 > 0.968, found to be degraded, and lycopene percentage was calculated. *Escherichia coli* colonies protected from UV rays by lycopene film were counted after different intervals (24, 48, and 72 h). The effect of UV rays was measured in percentage at 10, 20, and 30 min of exposure to UV rays. Sample *S. lycopersicum* had the highest UV‐ray blocking potential, that is, 99.75%, and sample *C. annuum* had the lowest UV‐ray blocking percentage, that is, 77.78%. Further research and development will be required to determine its effectiveness, stability, and practicality for creating lycopene films as a barrier against UV rays.

## Graphical abstract

*Solanum lycopersicum* had the highest (3.36024 μg/mL), whereas *Capsicum annuum* (0.31824 μg/mL) had the lowest lycopene concentration. *S. lycopersicum* lycopene showed the highest UV‐ray blocking efficiency, inhibiting 99.75% of UV rays after 72 hours. Lycopene shows significant potential as a UV protectant in bacterial cultures, suggesting it could be used in skincare products.

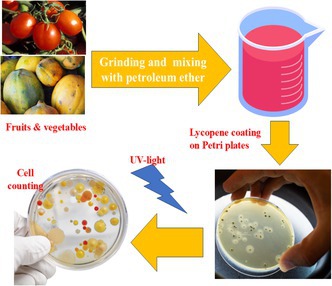

## 1 Introduction

Herbal‐based products can be used at a much higher level in the future due to accessibility, affordability, and global importance, with little or no side effects compared with conventional drugs (Ahmed et al. 2023). There is long‐standing scientific evidence that a diet rich in fruits and vegetables is good for health and a long life (Rosell and Fadnes 2024). In this context, fruits and vegetables are being used in cosmetics, which have been linked in many clinical trials to various types of environmental changes, including helping in preventing many skin‐related diseases like sunburn, etc. (Fam et al. 2022; Jideani et al. 2021). To avoid this, scientists and doctors have formulated several types of skin creams and medicines by chemical synthesis method. The chemically synthesized pigments in skin cosmetics raise biocompatibility and safety issues (Goyal and Jerold 2021; Prajapati et al. 2025). Moreover, the use of chemically synthesized cream for a longer time may cause many side effects. Recently, herbal‐based creams have gained popularity around the globe, which have almost no side effects (Jideani et al. 2021). Keeping this in mind, today, scientists or pharmacists across the world are continuously trying to formulate phytochemical‐based medicines and skin care creams that can protect humans from skin diseases caused by sunburn. Some of the plants and their fruits contain specific types of biomolecules that give the fruit a special color through the production of red pigments, which are responsible for the color pigments found in long hydrocarbon chains known as lycopene (dark red sticky liquid) (Lu et al. 2021).

Lycopene (C_40_H_56_:536.85 MW) is an unsaturated hydrocarbon carotenoid containing 13 carbon–carbon double bonds. Out of these 13 double bonds, 11 are conjugated and arranged in a linear array (Imran et al. 2020). This conjugated double bond in lycopene is responsible for the vibrant red color of the fruits (Bin‐Jumah et al. 2022). The red pigment of lycopene is a C_40_ carotenoid composed of eight isoprene units, making it a tetraterpene. Lycopene also serves as an intermediate for the biosynthesis of other carotenoids. It is found in moderate to high concentrations in fruits, such as peppers, papaya, tomatoes, watermelons, red grapes, and guavas 2021. From the various pieces of literature, it has been found that lycopene is freely soluble in acetone and petroleum and partially soluble in ethanol and acetone. Lycopene is a water‐insoluble, and the soluble solutions of lycopene in water or acetone and petroleum exhibit maximum absorbance at a wavelength (*λ*) around 503 nm (Villaseñor‐Aguilar et al. 2021). Lycopene extracts from fruits are used as a food coloring in dairy products, non‐alcohol‐flavored beverages, cereals, grain products, bread crumbs, and baked goods and spreads, imparting yellow to red color. Besides this, the lycopene molecules have good ray‐reflecting properties, which may block UV rays. From the literature, it has been proven that lycopene has several therapeutic values, including protection from UV rays, anticancer activity, antimicrobial activity, and heart‐related diseases (Rodríguez‐Negrete et al. 2024; Rudzińska et al. 2023).

There are several fruits and vegetables that are rich in lycopene. Lycopene in fruit products consists mostly of trans‐lycopene, accounting for 35%–96% of the total lycopene content, and low levels of cis‐lycopene, accounting for 1%–22% of the total lycopene content 2021. Some of the fruits that are rich in lycopene are *Capsicum annuum* (capsicum) (Alonso‐Villegas et al. 2023), *Capsicum frutescens* (chili) (Castro‐Muñoz et al. 2022), *Carica papaya* (papaya) (Ayodipupo Babalola et al. 2024; Kong et al. 2021), *C. lanatus* (watermelon) (Benmeziane and Derradji 2023; Sirikhet et al. 2021), and *Solanum lycopersicum* (tomato) (Benmeziane and Derradji 2023; Carvalho et al. 2021; Tran and Nguyen 2023). All these fruits and vegetables have medicinal value. For instance, *C. annuum* has been used to prevent stomach upset, toothache, poor circulation, fever, hyperlipidemia, and heart disease (Jang et al. 2020). Capsicum can also be used in the treatment of painful osteoarthritis, shingles, rheumatoid arthritis, postherpetic neuralgia, trigeminal neuralgia, diabetic neuropathy, fibromyalgia, muscle cramps, and back pain (Catalfamo et al. 2022). *C. frutescens* has preventive and therapeutic properties for cancer, arthritis, joint stiffness, bronchitis, and heart arrhythmia headache, and stomach diseases (Maya et al. 2021). Dietary antioxidants in chillies protect against many diseases, such as cancer and diabetes (Bal et al. 2022). Peppers have antimicrobial properties that are very important for human health. Also, standard capsaicin, medicinally prepared gels, creams, ointments, and essential oil from the pods have medicinal properties for treating many diseases (Füchtbauer et al. 2021). *C. papaya* is a natural medicine used for cancer, inflammation, aging, skin treatment, and lifelong diseases (Ayodipupo Babalola et al. 2024). Various scholars have highlighted the medicinal benefits of papaya extract and its phytochemicals (Ayodipupo Babalola et al. 2024; Hariono et al. 2021). *C. lanatus* , watermelon, is popular in folk medicine and has antimicrobial, antioxidant, anti‐plasmodial, anti‐inflammatory, anti‐prostatic hyperplasia activity, analgesic properties, antidiabetic, antiallergenic, and hepatoprotective activities (Elsayed et al. 2022; Sulieman and Ibrahim 2022). The seeds are used in the treatment of urinary tract infections, polyuria, such as bed‐wetting, ascites, and kidney stones, alcohol poisoning, high blood pressure, diabetes, diarrhea, and gonorrhea (Wahid and Saqib 2022). The fruits of *S. lycopersicum* are used as first aid for burns, scabs, and sunburn (Chidambaram et al. 2022; Raza et al. 2022). Tomatoes are rich in lycopene, which benefits the heart, prostate, arthritis, and headache. Its root provides relief from toothache. A certain mixture of its leaves in castor oil is used to treat leprosy (Collins et al. 2022).

**FIGURE 1:**
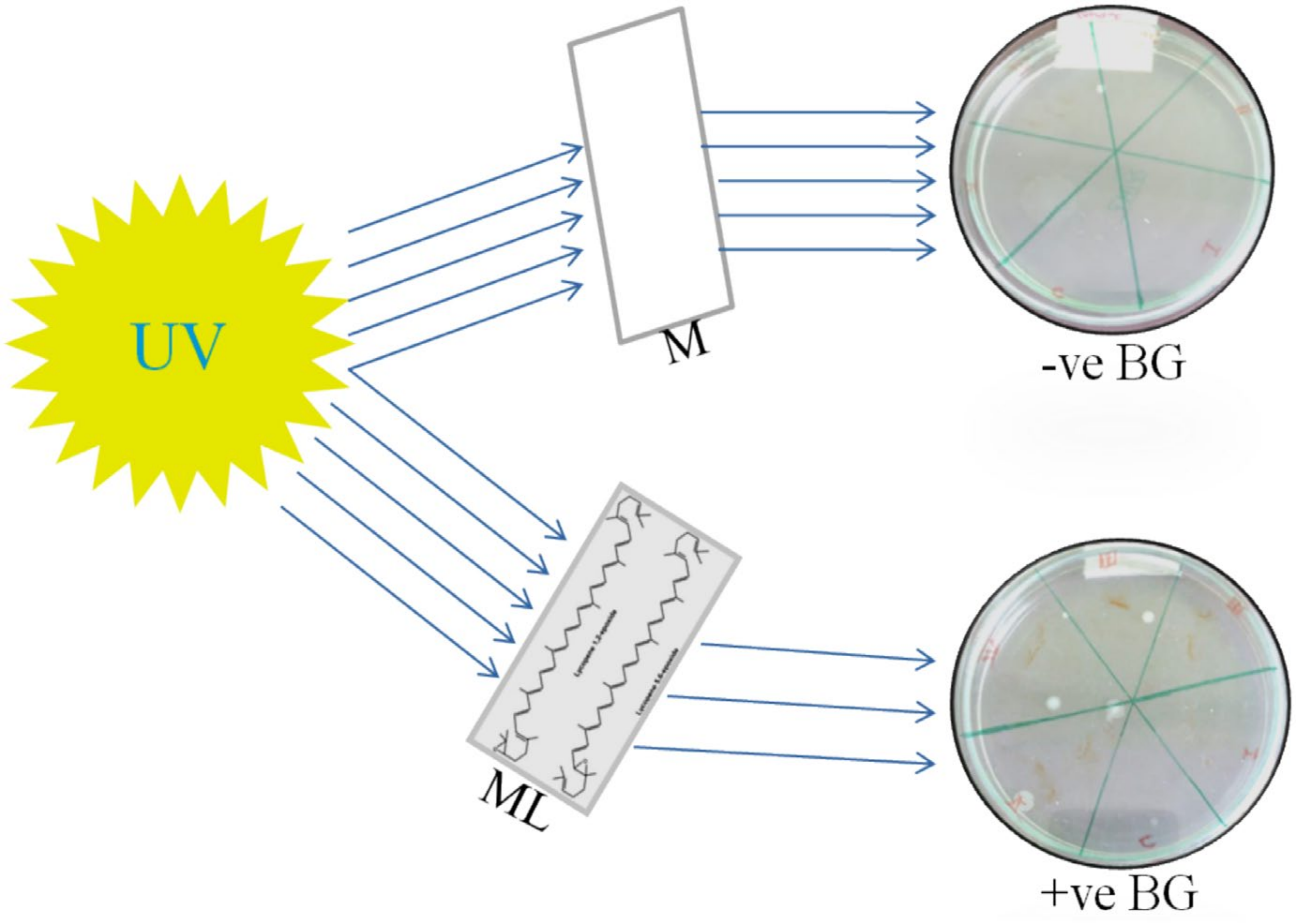
UV‐ray's effect on the bacterial culture of *Escherichia coli* with the filters of normal glass and lycopene‐coated glass (Çavuşoğlu et al. 2021).

The effect of the antimicrobial activity of lycopene compounds against some pathogens, such as bacteria and fungi, was analyzed. There are several investigations where investigators have used the isolated lycopene from the above fruits and used it to inhibit the growth of microorganisms. Çavuşoğlu et al. (2021). assessed the ability of lycopene against UV rays by dividing groups of five fruits treated with lycopene. It was found that UV‐ray irradiation affected all the parameters examined, whereas the UV rays mediated anti‐inflammatory activities of lycopene were dependent on the concentration of lycopene. Therefore, the study demonstrated the promising ability of lycopene to protect bacterial cultures against the harmful effects of UV‐ray exposure (Figure 1). There is growing evidence that lycopene may protect human skin from the harmful effects caused by solar ultraviolet (UV) radiation. Accordingly, based on the results of previously conducted studies, a meta‐analysis showed that supplementation with lycopene is associated with protection against sunburn creams and lotion development.

Gupta et al. (2013) isolated the lycopene from the tomato (*Lycopersicum esculentum*) and assessed its potential as a chemo‐preventive agent for hepatocarcinogenesis in mice. Here, the lycopene was extracted in hexane/acetone/ethanol and characterized by UV–visible, nuclear magnetic resonance (NMR), and Fourier transform infrared spectroscopy. The mice study exhibited that there was a reduction in tumor incidence (42.05%), tumor burden (1.39), and tumor multiplication (3.42) upon LycT pretreatment of NDEA‐treated animals.

Based on the above study, lycopene extracted from the fruits was evaluated against Gram‐negative bacteria, that is, *E. coli* , by making a thin film of lycopene on the bottom surface of the Petri plates. Current research focuses on some selected fruit extracts that have been used to protect or reflect harmful sun rays on the skin and to protect the skin from sunburn.

The primary aim of this research work was to extract the lycopene from the various fruit pulps. One of the objectives was to perform a qualitative analysis of the lycopene yield from the fruit. Another objective was to characterize the lycopene by using various characterization techniques. Finally, under different experimental conditions, the UV‐blocking activity of different lycopenes was assessed against Gram‐negative bacteria, that is, *Escherichia coli* . It was hoped that lycopene extracted from selected fruits would be used in cosmetic products, such as creams and gels to protect against harmful UV rays.

## 2 Materials and Methods

### 2.1 Materials

Selected fruits, such as *Capsicum annuum* (capsicum), *Capsicum frutescens* (chili), *Carica papaya* (papaya), *Citrullus lanatus* (watermelon), and *Solanum lycopersicum* (tomato), were procured from the local market. All these fruits were coded as *Capsicum annuum* (*C.a*.), *C. frutescens* (*C.f*.), *C. papaya* (*C. p*.), *C. lanatus* (*C. l*.), and *Solanum lycopersicum* (*S. l*.), respectively, and used freshly for the experiment: acetone (Merck, Mumbai, India), petroleum ether (Himedia, Mumbai, India), double distilled water, Eosin methylene blue (EMB) media (Himedia, Mumbai, India), separating funnel (Bosrosil), and *Escherichia coli* (Stock from laboratory). All the chemicals and media were AR grade with high purity.

#### 2.1.1 Solvents Selection

Polar aprotic acetone was used as an aprotic polar solvent for the extraction and solubilization of both hydrophilic and hydrophobic molecules of total phytochemicals, including lycopene. The polar protic was hydrolyzed to 5% sodium sulfate in a protic solvent that favored the water‐like bimolecular mechanism responsible for dissociating hydrophilic biomolecules. Nonpolar petroleum ether was used as a nonpolar solvent for the separation of soluble and lipids, which was found to have a special ability to dissolve a particular series of hydrocarbon molecules and lycopene (Kyriakoudi et al. 2022; Zuorro 2020).

#### 2.1.2 Sample Preparation

**FIGURE 2:**
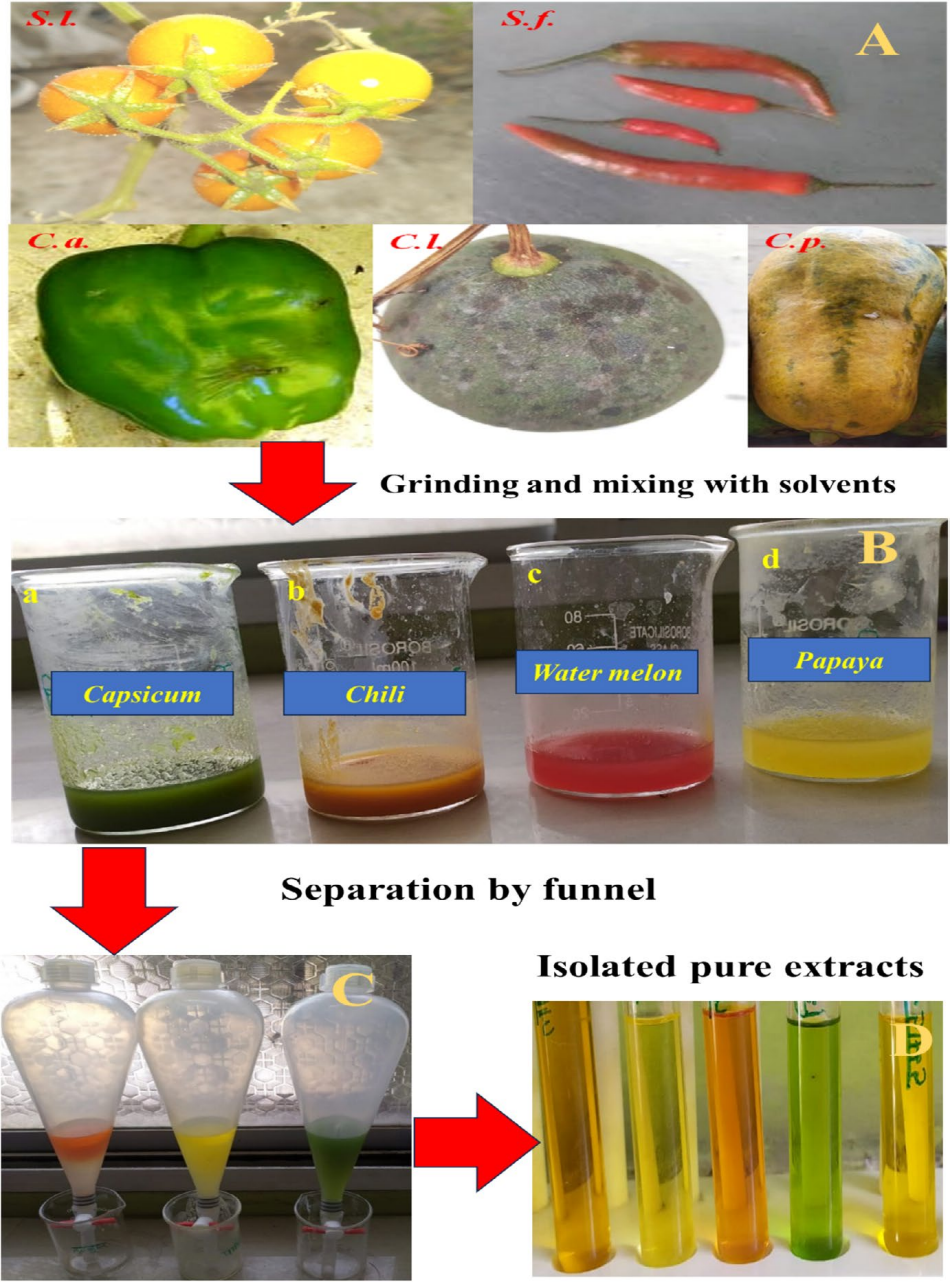
Steps involved in the extraction of purified lycopene from the fruits. (A) grinding of the fruits, (B) mixing with solvents, (C) separation of the compound with the separating funnel, (D) purified lycopene from all the fruits. Fruits for extraction of lycopene were coded as *Solanum lycopersicum* (*S. l*.), *C. frutescens* (*C.f*.), *C. annuum* (*C.a*.), *C. lanatus* (*C. l*.), and *C. papaya* (*C. p*.)

The fruits' pulp was cut separately into small pieces, approximately 2–3 cm cubes. All five fruit samples cut individually were weighed on a weighing balance. Furthermore, each fruit pulp was placed separately in a mortar and pestle and ground finely. Additionally, acetone was added to each pulp during the grinding. During this process, the entire pulp was repeatedly taken out with acetone until the residue became colorless. Furthermore, the acetone extract of each fruit was collected and transferred separately to a separate funnel. To each separating funnel, approximately 20 mL of petroleum ether and acetone extract was added gently. Furthermore, approximately 20 mL of 5% NaSO_4_ solution was added, and the separating funnel was shaken gently. During this process, the volume of petroleum ether was reduced because of evaporation, so 20 mL of more petroleum ether was added to each separating funnel to separate the two layers clearly. Most of the color will appear in the petroleum ether layer. Both phases were separated, and the lower aqueous phases were re‐extracted with an additional 20 mL of petroleum ether until the aqueous phases became colorless. The petroleum ether extract was collected and washed once with double‐distilled water. Furthermore, the washed petroleum ether extract containing lycopene was transferred to an amber bottle (Khongthaw et al. 2024). The detailed steps involved in extracting lycopene from the fruit samples are given in Figure 2. The pH value plays an important role in determining the potential number of hydrogens in lycopene‐containing samples in an anhydrous liquid (Khan et al. 2021). The pH values of the lycopene samples extracted in petroleum ether at 20°C were determined using a pH meter.

#### 2.1.3 Analysis of Quantity and Quality of Lycopene

**FIGURE 3:**
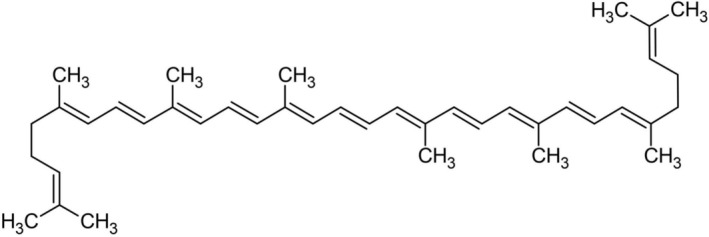
Chemical structure of lycopene.

The absorbance maxima of the lycopene were found to be 503 nm (*λ*) by UV–Vis instruments. So, the measurement was taken at this particular wavelength while the petroleum ether was used as a reference sample. The light path up to 1 cm gives an absorption of 17.2 × 10^4^/M × cm. Therefore, a concentration of 3.1206 μg lycopene/mL gives unit absorbance (Desai et al. 2018). Figure 3 shows the structure of lycopene (Suwanaruang 2016).

#### 2.1.4 UV‐Ray Inhibition Activity of Lycopene on *E. coli*

Firstly, the upper lid's sterilized Petri plate bottom surface was thoroughly coated with the lycopene extract. The following concentrations of lycopene (1, 2, 3, 4, and 5 mL) were used for coating each lid separately. Before this, lycopene concentrations were evaporated to a final volume of 1 mL, which was used with 1 mL of 10% starch. Further sterilized Eosin methylene blue media (EMB) was poured on the lower part of the Petri plate and allowed to solidify. Furthermore, once the media was solidified, the *E. coli* sample was spread on the agar surface with the help of a micropipette and a glass spreader by using the spread plate technique. This was followed by exposure to UV rays to each plate for 10, 20, and 30 min. The treated plates were placed in an incubator for 24, 48, and 72 h at 37°C to allow the bacteria to grow. Furthermore, after a specific time, all the pewter plates were taken out from the incubator, and bacterial colonies were counted, comparing the effect of UV rays on all the plates without UV rays (control) (Ascenso et al. 2016; Cooperstone et al. 2017; Fazekas et al. 2003).

#### 2.1.5 Statistical Procedures

The prescribed standard statistical methods analyzed the data collected during the study. The parameters of the samples were presented in terms of mean ± SD. Descriptive statistics were maintained while the acceptability of the data obtained was determined by analyzing their regression coefficients through the *Y* value and the *R* ^2^value ranging from 0 to 1, indicating how well the model can predict the outcome of the dependent variable. The *R* ^2^ value of 0 means the percentage of the relationship between the dependent and independent variables, which the model explained. Graphical representation was done using Microsoft Excel software.

## 3 Results and Discussion

Lycopene, a natural pigment found in fruits and vegetables, can absorb UV rays because of its chemical structure. Lycopene is primarily known for its antioxidant properties and potential health benefits when consumed internally (Bin‐Jumah et al. 2022). This could potentially use lycopene, a helpful ingredient in sunscreens or skin care products designed to protect against UV rays. Optimizing lycopene extraction from selected fruits and its use for protection against UV rays, focusing on bacteria ( *E. coli* ), was attempted. Factors, such as the type of fruit, extraction method, and solvent play an important role in optimizing lycopene extraction. Previously, different fruits were treated using different extraction techniques to obtain the maximum amount of lycopene. Furthermore, a critical method is used to preserve the stability of lycopene and its beneficial properties during extraction. When lycopene was used to protect against UV rays (Bin‐Jumah et al. 2022), its antioxidant properties were found to help neutralize free radicals produced by UV‐ray exposure, which may reduce skin damage. Additionally, lycopene has been shown to have some antibacterial properties that protect against bacterial infections.

**FIGURE 4:**
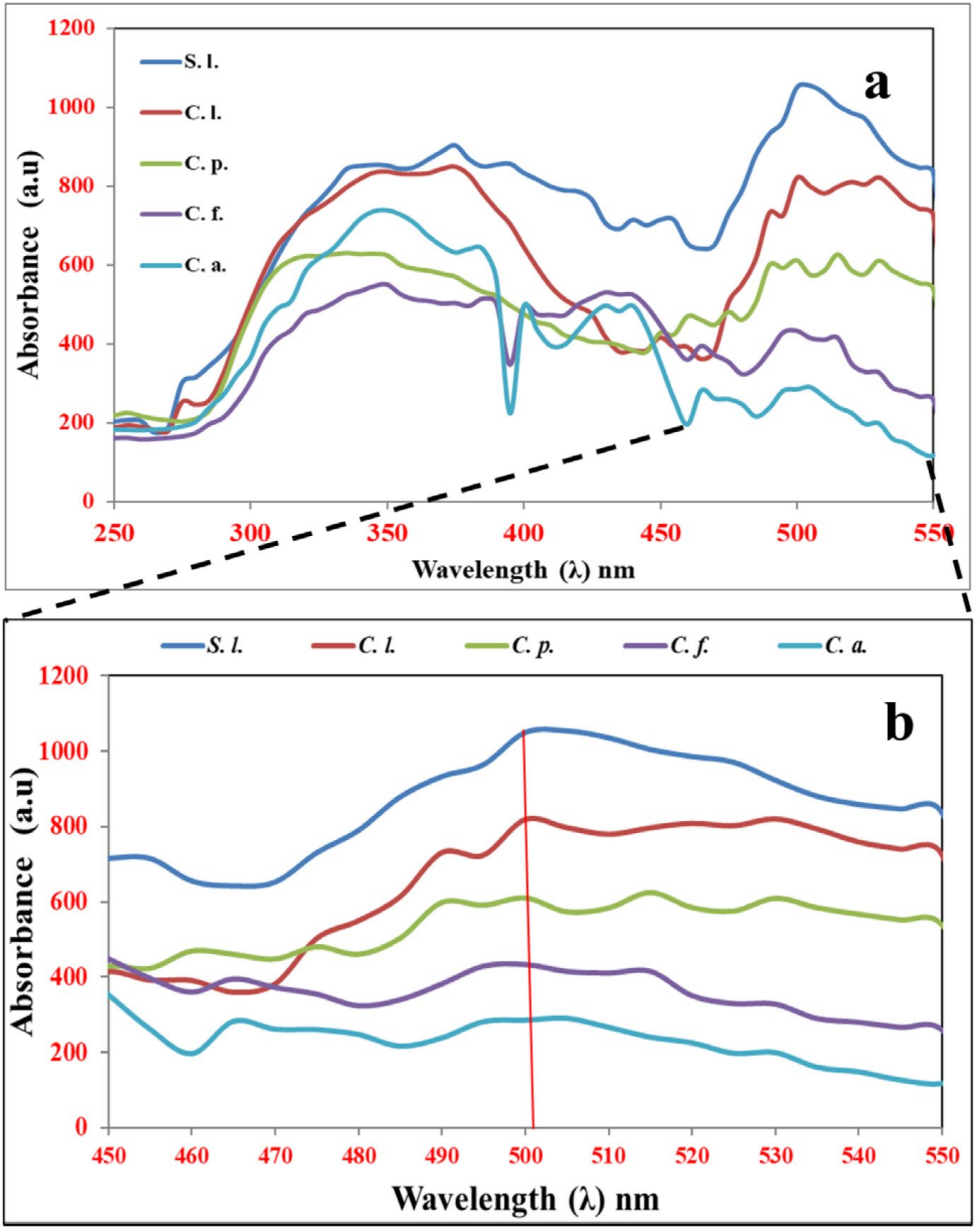
UV–Vis. spectra of lycopene extracted from different fruit samples (a) scanning and (b) at *λ*_503_ nm.

Analysis of lycopene‐containing samples were analyzed UV–Vis spectra of lycopene obtained at the selected wavelength of 503 nm were determined to determine the lycopene concentration extracted from fruits in selected organic solvents. The highest absorbance spectra peak was found at 505 nm in *S. lycopersicum* ., 375 nm in *C. lanatus* (*C. l*.), 515 nm in *C. papaya* (*C. p*.), 350 nm in *C. frutescens* (*C.f*.), and *C. annuum* (*C.a*.), as shown in Figure 4a,b. The absorbance peak at 503 nm was used to analyze and determine the concentration of lycopene in the samples. According to the results obtained, spectra between 450 nm and 550 nm were analyzed to estimate expected lycopene concentrations, where the highest absorbance peak was between 500 and 510 nm wavelength, which was suitable for the analysis of lycopene molecules present in the liquid medium. The concentrations of lycopene present in the samples were arranged in ascending order from lowest to highest concentration as (*C.a*.) < (*C. f*.) < (*C. p*.) <(*C. l*.) <(*S. l*.), the details are shown in Figure 4b. Lycopene content was measured in a spectrophotometer at 503 nm (*λ*). A light path of up to 1 cm gives an absorption of 17.2 × 10^4^/M×cm. Therefore, the concentration of lycopene in 3.1206 μg/mL gives unit absorbance. The results indicate that S.l. had the highest amount of lycopene, that is, 3.36024 μg/mL, whereas *C. annuum* (C.a.) had the lowest, that is, 0.31824 μg/mL.

The UV–Vis absorbance peak of lycopene extracted from all the fruits and vegetable samples occurs in the 440–530 nm range (Table 1). This suggests a π‐π* electronic transition associated with the HOMO‐LUMO frontier orbitals localized in the double bonds in lycopene (Numan et al. 2022).

**TABLE 1:** Comparison of UV Vis absorbance peaks of lycopene from different fruits in the present investigation and existing literature.

Present investigation		Previous study	References
Samples	Absorbance peak (nm)	Absorbance peak (nm)	
Tomato ( *Solanum lycopersicum* )	505	468 444, 470 (maxima) and 500 445, 472 and 503	Nair and Lilwani (2016), Popescu et al. (2022), Ranveer et al. (2020)
Watermelon ( *C. lanatus* )	375	459 443.2, 472 (maxima) and 503	(Nair and Lilwani (2016), Okonkwo et al. (2018)
*C. annuum*	350	450, 275 small peak, 400	(“Final Report on the Safety Assessment of *Capsicum annuum* Extract, *Capsicum annuum* Fruit Extract, *Capsicum annuum* Resin, *Capsicum annuum* Fruit Powder, *Capsicum frutescens* Fruit, *Capsicum frutescens* Fruit Extract, *Capsicum frutescens* Resin, and Capsaicin1,” 2007), Richins et al. (2014)
*C. frutescens*	350	—	Davis et al. (2003)
*C. papaya*	515	440, 450–500	Rayhan et al. (2019)

**FIGURE 5:**
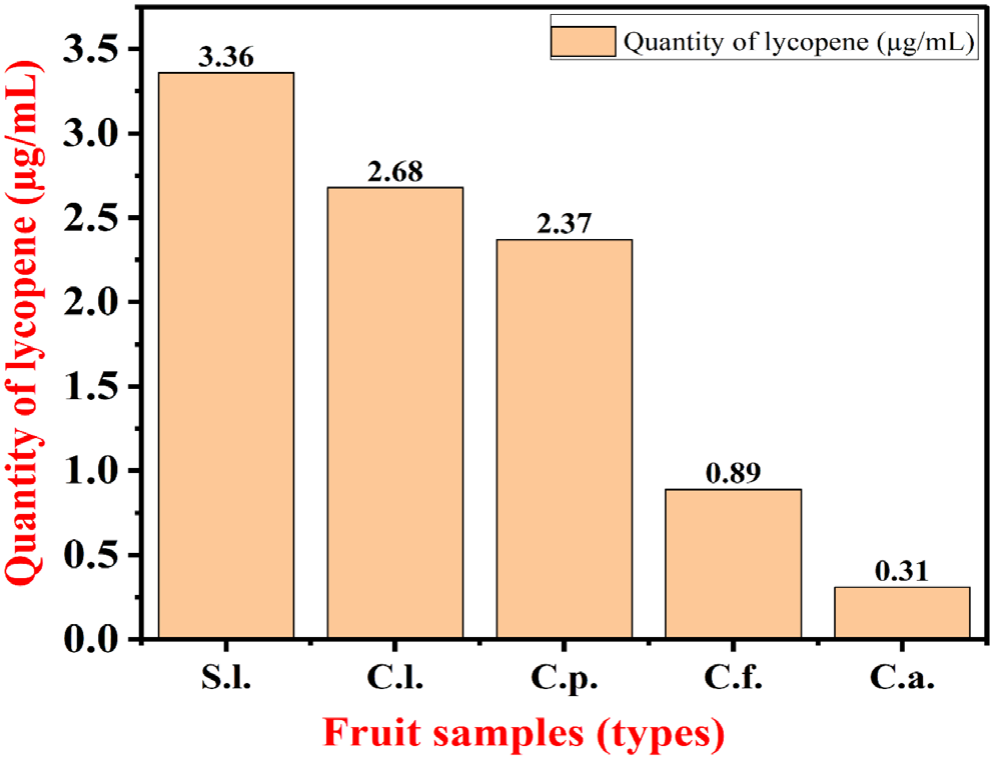
Lycopene quantities in μg/mL from different fruits.

Figure 5 shows that the sample of *S. lycopersicum* (*S.l*.) was richer in lycopene material than other samples.

**FIGURE 6:**
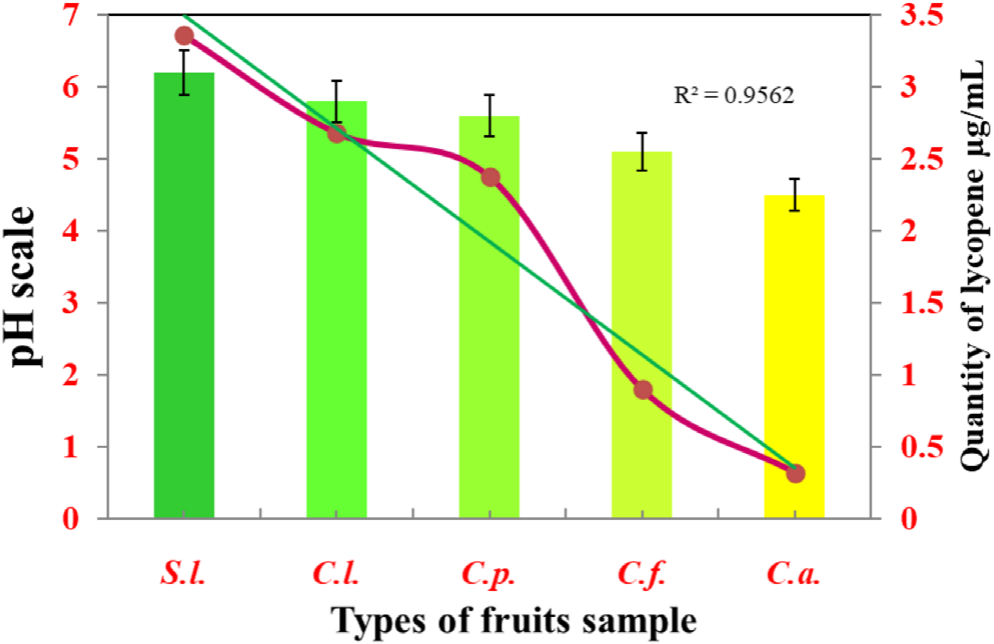
pH and lycopene quantities in μg/mL from different fruit samples.

The pH value of lycopene extracted from different fruits was found in the range of 0–7 with some standard errors. The sample of *S. lycopersicum* had pH values of 6.2 and a maximum quantity of 3.36024 μg/mL of lycopene. The sample of *C. annuum* (*C.a*.) was found to have pH values of 4.5 and a minimum amount of lycopene at 0.31824 μg/mL. Other samples, *C. lanatus* , *C. papaya* , and *C. frutescens* , were found with pH values and content of lycopene quantities between the samples *S. lycopersicum* and *C. annuum* (*C.a*.) samples. The *R* ^2^ value of the correlation variables between pH value and lycopene content was found to be 1 > 0.956 from the samples (*S. l*., *C. l*., *C. p*., *C. f*., and *C. a*.), Figure 6.

**FIGURE 7:**
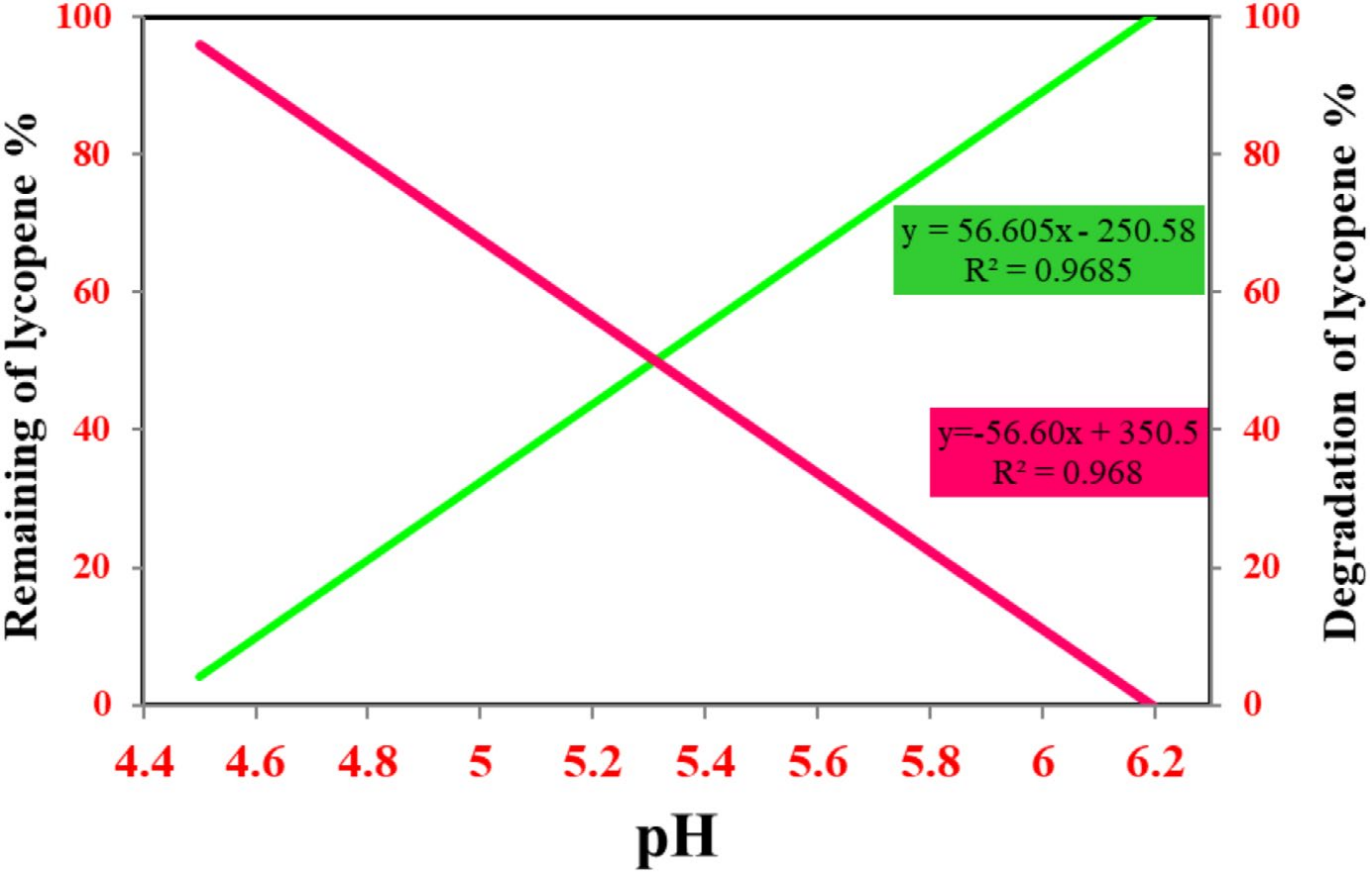
pH effect on the remaining and degradation of lycopene percentage.

The *R* ^2^ of the obtained data measured or predicted the proportion of variation in the dependent variable explained by the independent variable in the data expected from the pH values of lycopene extracted from different fruits was not considered falsely optimistic. This was on a scale of 0–1, where 0 indicated that the model did not explain any of the variability and 1 showed that it explained all the variables. The pH value was increased with good quality in sufficient quantity of lycopene extracted from different fruits, known as residual lycopene percentage. The interrelated variables between pH value and lycopene percentage were found using the equation *y* = 56.60×−250.5, and their *R* ^2^ value was close to 1 > 0.968. The variables between pH value and degradation lycopene percentage were found using the equation *y* = −56.60×+250.5, and their *R* ^2^ value was found to be close to 1 > 0.968, which was optimistically estimated; the dependent variable was obtained from the pH analysis of lycopene, and data were explained by the independent variables, Figure 7.

**FIGURE 8:**
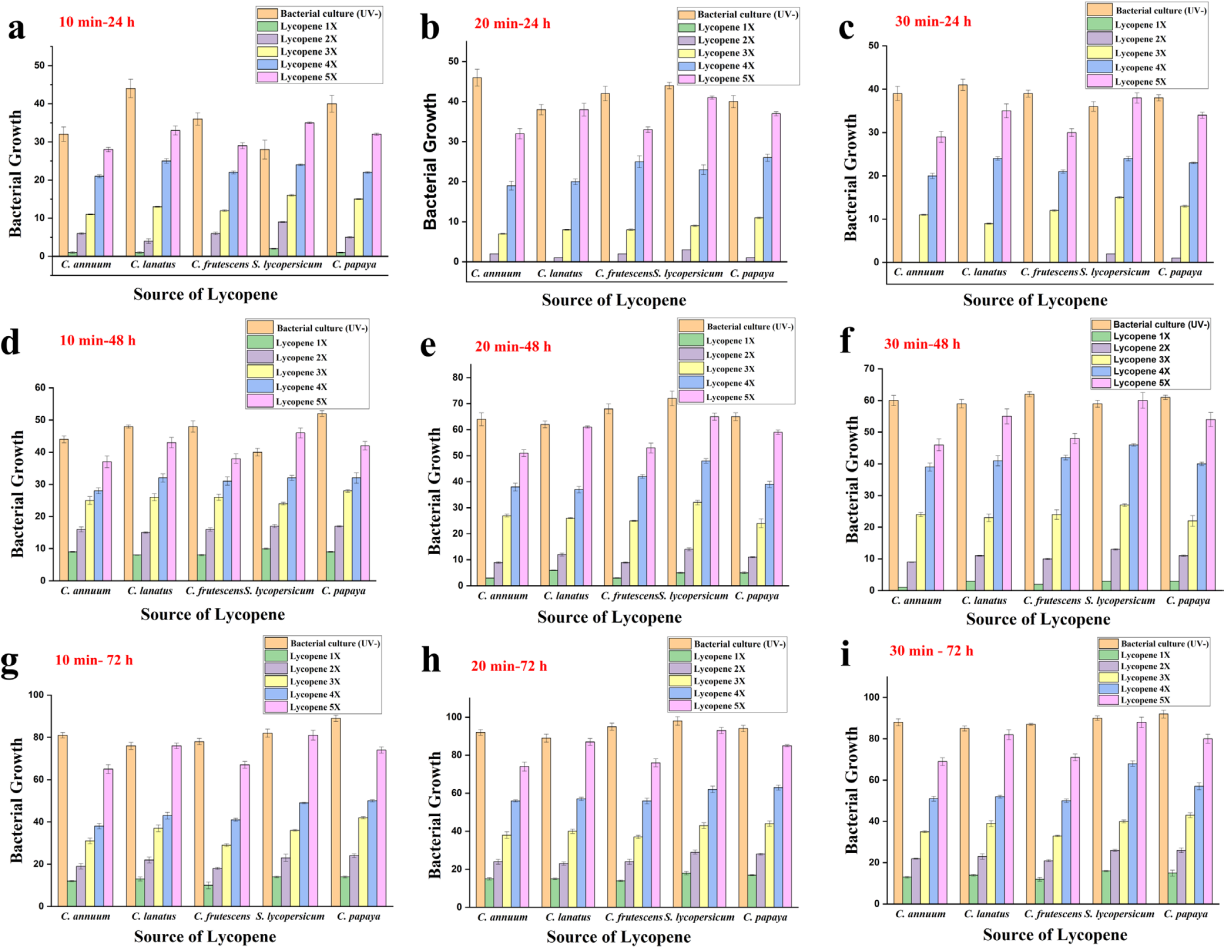
Effect of UV exposure time on the lycopene‐coated bacterial culture plates grown after 24 h and 37°C: (a) 10 min, (b) 20 min, and (c) 30 min. Bacterial growth after 24 h, (d) 10 min, (e) 20 min, and (f) 30 min and bacteria grown after 72 h, (g) 10 min, (h) 20 min, and (i) 30 min.

The results of all the plates showing the lycopene coating as a barrier for UV rays are shown in Tables 2, 3, 4, 5, 6, 7, 8, 9, 10 and Figure 8a–i, after different exposure times of UV and incubation times of *E. coli* .

**TABLE 2:** Bacterial culture: UV rays exposed for 10 min and incubated at 37°C for 24 h.

Extraction of lycopene from fruit samples	Bacterial culture (UV−)	Bacterial culture (UV+)	Lycopene concentrations to prevent UV rays				
			1×	2×	3×	4×	5×
*C. annuum*	32	0	1	6	11	21	28
*C. lanatus*	44	0	1	4	13	25	33
*C. frutescens*	36	0	0	6	12	22	29
*S. lycopersicum*	28	0	2	9	16	24	**35**
*C. papaya*	40	0	1	5	15	22	32

*Note:* The bold value indicates the highest value only.

**TABLE 3:** Bacterial culture, UV rays exposed for 20 min and incubated at 37°C for 24 h.

Extraction of lycopene from fruit samples	Bacterial culture (UV−)	Bacterial culture (UV+)	Lycopene concentrations to prevent UV rays				
			1×	2×	3×	4×	5×
*C. annuum*	46	0	0	2	7	19	32
*C. lanatus*	38	0	0	1	8	20	38
*C. frutescens*	42	0	0	2	8	25	33
*S. lycopersicum*	44	0	0	3	9	23	**41**
*C. papaya*	40	0	0	1	11	26	37

*Note:* The bold value indicates the highest value only.

**TABLE 4:** Bacterial culture: UV rays exposed for 30 min and incubated at 37°C for 24 h.

Extraction of lycopene from fruit samples	Bacterial culture (UV−)	Bacterial culture (UV+)	Lycopene concentrations to prevent UV rays				
			1×	2×	3×	4×	5×
*C. annuum*	39	0	0	0	11	20	29
*C. lanatus*	41	0	0	0	9	24	35
*C. frutescens*	39	0	0	0	12	21	30
*S. lycopersicum*	36	0	0	2	15	24	**38**
*C. papaya*	38	0	0	1	13	23	34

*Note:* The bold value indicates the highest value only.

**TABLE 5:** Bacterial culture: UV rays exposed for 10 min and incubated at 37°C for 48 h.

Extraction of lycopene from fruit samples	Bacterial culture (UV−)	Bacterial culture (UV+)	Lycopene concentrations to prevent UV rays				
			1×	2×	3×	4×	5×
*C. annuum*	44	0	9	16	25	28	37
*C. lanatus*	48	0	8	15	26	32	43
*C. frutescens*	48	0	8	16	26	31	38
*S. lycopersicum*	40	0	10	17	24	32	**46**
*C. papaya*	52	0	9	17	28	32	42

*Note:* The bold value indicates the highest value only.

All plates, including the control (UV−), were incubated at 37° for 24 h for bacterial growth. Furthermore, the colonies on the non‐UV‐treated bacterial plates were compared with the test plates (bacterial colonies protected from UV rays by a lycopene film). In the case of plates exposed to 10 min of UV rays, the plates coated with lycopene film (5×) obtained from *S. lycopersicum* exhibited the best results, that is, as there was a maximum number of *E. coli* colonie*s*, that is, 35. The lowest number of *E. coli* colonies was present with the lycopene coating obtained from *C. annuum* , which was 28. This indicated that lycopene obtained from sample *S. lycopersicum* was found to have more ability to block UV rays whereas *C. annuum* has the least (Figure 8a–c). Similarly, *E. coli* colonies were counted after different incubation time intervals, that is, 48 (Figure 8d–f) and 72 h (Figure 8g–h), at the same temperature of 37°C. Here, the lycopene coating from *S. lycopersicum* showed the best result, that is, 46 and 81 colonies after 48 and 72 h, respectively. The lowest number of *E. coli* colonies, that is, 38 and 65, was found in *C. annuum‐extracted* lycopene coating on the plates for 48 and 72 h, respectively. Similarly, even with different UV‐ray exposure times of 20 and 30 min, the most effective UV‐ray blocking effect was seen with lycopene obtained from sample *S. lycopersicum* . In contrast, the least effect was obtained from sample *C. annuum* . Therefore, the impact of blocking UV rays was seen in all types of lycopene‐containing materials.

**TABLE 6:** Bacterial culture: UV rays exposed for 20 min and incubated at 37°C for 48 h.

Extraction of lycopene from fruit samples	Bacteria culture (UV−)	Bacteria culture (UV+)	Lycopene concentrations to prevent UV rays				
			1×	2×	3×	4×	5×
*C. annuum*	64	0	3	9	27	38	51
*C. lanatus*	62	0	6	12	26	37	61
*C. frutescens*	68	0	3	9	25	42	53
*S. lycopersicum*	72	0	5	14	32	48	**65**
*C. papaya*	65	0	5	11	24	39	59

*Note:* The bold value indicates the highest value only.

**TABLE 7:** Bacterial culture: UV rays exposed for 30 min and incubated at 37°C for 48 h.

Extraction of lycopene from fruit samples	Bacterial culture (UV−)	Bacterial culture (UV+)	Lycopene concentrations to prevent UV rays				
			1×	2×	3×	4×	5×
*C. annuum*	60	0	1	9	24	39	46
*C. lanatus*	59	0	3	11	23	41	55
*C. frutescens*	62	0	2	10	24	42	48
*S. lycopersicum*	59	0	3	13	27	46	**60**
*C. papaya*	61	0	3	11	22	40	54

*Note:* The bold value indicates the highest value only.

**TABLE 8:** Bacterial culture: UV rays exposed for 10 min and incubated at 37°C for 72 h.

Extraction of lycopene from fruit samples	Bacterial culture (UV−)	Bacterial culture (UV+)	Lycopene concentrations to prevent UV rays				
			1×	2×	3×	4×	5×
*C. annuum*	81	0	12	19	31	38	65
*C. lanatus*	76	0	13	22	37	43	76
*C. frutescens*	78	0	10	18	29	41	67
*S. lycopersicum*	82	0	14	23	36	49	**81**
*C. papaya*	89	0	14	24	42	50	74

*Note:* The bold value indicates the highest value only.

Different types of fruits contained different amounts of lycopene and different nutritional values. Lycopene molecules were found to have potential properties as a blocker of UV rays. This was measured in percentage over a certain time interval, such as 10, 20, and 30 min of exposure to UV rays. The effect of UV rays on bacterial culture was analyzed for different incubation periods. According to the UV‐ray blocking properties of lycopene extracted from some selected fruits, their abbreviated names were written as (*C.a*., *C.l*., *C.f*., *S. l*., and *C.p*.). The analyzed aspect values found that the sample *S. lycopersicum* had the highest 99.75% UV‐ray blocking effect at the optimum time of 72 h, and the sample *C. annuum* (*C.a*.) had the lowest UV‐ray inhibition of 77.78% was given from 24 h after 10 min of exposure to UV rays on bacterial culture. The analyzed aspect values found that the sample *S. lycopersicum* had the highest 99.55% UV‐ray blocking effect given at the optimum time of 72 h, and the sample *C. annuum* (*C.a*.) had the lowest UV‐ray inhibition of 75.13% was given from 24 h after 30 min of exposure to UV rays on bacterial culture. The analyzed aspect values found that the sample *S. lycopersicum* had the highest 99.36% UV‐ray blocking effect at the optimum time of 72 h. The sample *C. annuum* (*C.a*.) had the lowest UV‐ray inhibition of 76.19% from 24 h after 20 min of exposure to UV rays on bacterial culture.

**FIGURE 9:**
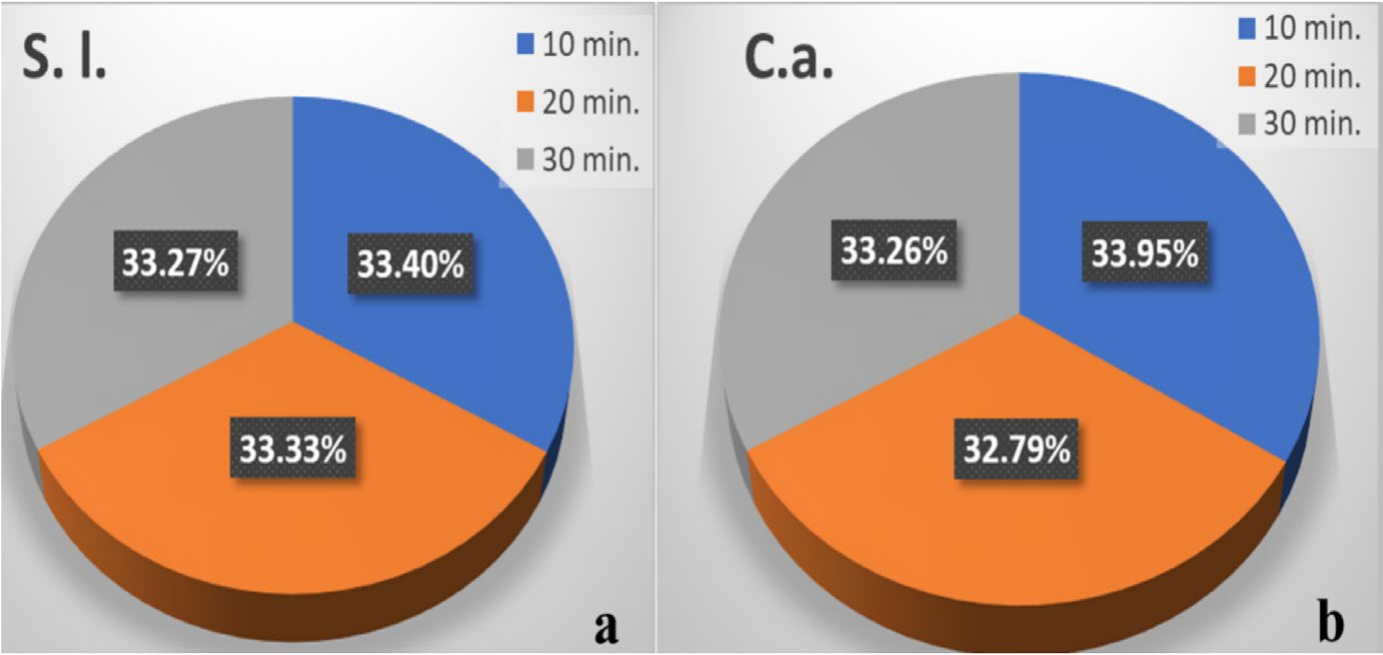
Comparative aspect of UV‐ray inhibition percentage of lycopene obtained from *S.l*. (a) and (b) and *C.a*., for the exposure time of 10, 20, and 30 min on bacterial culture.

Lycopene obtained from *S. lycopersicum* was used to block the UV rays and promote bacterial growth after 72 h. To observe the blocking effect, the *E. coli* was exposed to UV rays for 10, 20, and 30 min. The highest blocking of UV rays was 33.40% when the *E. coli* was exposed to 10 min of UV rays; for 20 min, it was 33.33%, and the lowest blocking of UV rays was observed in the case of 30 min of UV rays' exposure, which was 33.27%. Similarly, after 10, 20, and 30 min of UV exposure to *E. coli* , which had a coating of lycopene derived from *C. annuum* , were analyzed. After 10 min of UV exposure, the blocking percentage of UV after 24 h was the highest, that is, 33.95%, whereas the lowest was after 20 min of UV‐ray exposure, where the blocking percentage of UV rays was 32.79%. After 30 min of UV exposure, the blocking percentage of UV was 33.26%, as shown in Figure 9a,b.

**TABLE 9:** Bacterial culture: UV rays exposed for 20 min and incubated at 37°C for 72 h.

Extraction of lycopene from fruit samples	Bacterial culture (UV−)	Bacterial culture (UV+)	Lycopene concentrations to prevent UV rays				
			1×	2×	3×	4×	5×
*C. annuum*	92	0	15	24	38	56	74
*C. lanatus*	89	0	15	23	40	57	87
*C. frutescens*	95	0	14	24	37	56	76
*S. lycopersicum*	98	0	18	29	43	62	**93**
*C. papaya*	94	0	17	28	44	63	85

*Note:* The bold value indicates the highest value only.

**TABLE 10:** Bacterial culture: UV rays exposed for 30 min and incubated at 37°C for 72 h.

Extraction of lycopene from fruit samples	Bacterial culture (UV−)	Bacterial culture (UV+)	Lycopene concentrations to prevent UV rays				
			1×	2×	3×	4×	5×
*C. annuum*	88	0	13	22	35	51	69
*C. lanatus*	85	0	14	23	39	52	82
*C. frutescens*	87	0	12	21	33	50	71
*S. lycopersicum*	90	0	16	26	40	68	**88**
*C. papaya*	92	0	15	26	43	57	80

*Note:* The bold value indicates the highest value only.

**TABLE 11:** Previous studies on lycopene and other phytochemical extracts from various vegetables and fruits for antimicrobial activity.

Fruit type	Lycopene/pigment	Bacteria inhibited	MIC	MBC	References
*C. annum*	Methanol extract	*Staphylococcus aureus*	Not specified (NS)	NS	Ekom et al. (2021)
	Ethanolic extract	*Escherichia coli* (MDR)	9.4–37.5 mg/mL	18–37.5 mg/mL	Anikwe et al. (2017)
	Ethanolic extract	*E. coli* (MDS)	4.7–37 mg/mL	9.4–37 mg/mL	Anikwe et al. (2017)
	Ethanolic extract	*Listeria monocytogenes*	9.4–37.5 mg/mL	14.1–37.5 mg/mL	Anikwe et al. (2017)
*Capsicum frutescens*					
	Unfiltered extract	*Proteus mirabilis*	NS	NS	Isaac and Oluyomi (2020)
	Filtered extract	*Bacillus licheniformis*	NS	NS	Isaac and Oluyomi (2020)
	Capsaicin extract	*Salmonella*	No colonies observed, indicating strong inhibition	NS	Peeyananjarassri (2022)
	Var. fingerh	*Pseudomonas aeruginosa*	7.8	NS	Bello et al. (2015)
	Var. minima	*Salmonella typhi*	37.5	NS	Bello et al. (2015)
	Ethanol extract	*Staphylococcus epidermidis*	NS	NS	Husna et al. (2023)
	Capsaicin extract	*Staphylococcus aureus* , *E. coli* , *P. aeruginosa* , *S. typhi* and *Listeria monocytogenes*	NS	NS	Thapa Magar and Shrestha (2023)
*Carica papaya*	Papaya seed ethanol	*Propionibacterium acne*	NS	NS	Azizah et al. (2024)
	Papaya leaf ethanol	*Vibrio cholerae* , *S. typhi*	NS	NS	Utami et al. (2023)
	Papaya flower ethanol	*E. coli* ATCC 25922	2 mg/mL	NS	Anugraheni and Rini (2023)
	Papaya seed gel	Methicillin‐resistant *Staphylococcus aureus* (MRSA)	NS	NS	Riski et al. (2023)
*Citrullus lanatus*	Aqueous extract	*K. pneumoniae* *S. aureus*	0.195 0.30–0.39	0.39 0.78–2.73	Dewu et al. (2023)
	Methanol	*K. pneumoniae* , *S. aureus*	NS	NS	Dewu et al. (2023)
*Solanum lycopersicum*	Ethanol extract	*Streptococcus mutans* , *Porphyromonas gingivalis*	NS	NS	Rahmawati et al. (2023)
	Ethanol extract	*S. epidermidis*	NS	NS	Pauzan et al. (2023)

Buxani et al. (2016) also extracted phytochemicals from *C. frutescens* L using different solvent‐based extract methods. The antibacterial assay of the extracts revealed that *E. coli* , *P. aeruginosa* , *K. pneumoniae* , and *B. cereus* were sensitive to all the extracts tested. In contrast, none of the extracts showed inhibitory activity against *S. aureus* methicillin‐resistant *Staphylococcus aureus* (MRSA). The maximum ZOI was exhibited by the chloroform extract against *P. aeruginosa* (15.3 mm) and *K. pneumoniae* (13.5).

Lycopene, a natural pigment that can absorb UV light, holds significant potential as a UV protection agent. Its use in creating film barriers for direct UV protection is a promising application. However, further research and development are necessary to determine its effectiveness, stability, and practicality for creating lycopene films as a barrier against UV rays (Li and Yu 2023; Zhang et al. 2024). Factors, such as the concentration of lycopene in the film, its ability to adhere to surfaces, and its durability under UV exposure, will need to be considered. While using lycopene as a film for UV protection is intriguing, additional study and testing will be required to assess its feasibility and potential benefits. Performing more experiments or consulting experts in materials science and UV protection will provide valuable insights. Table 11 shows the lycopene and other phytochemical extracts from various vegetables and fruits for antimicrobial activity from previous studies.

Different fruit extracts, particularly from Capsicum, *Carica papaya* , and *Citrullus lanatus* , exhibit broad‐spectrum antibacterial activity against both Gram‐positive and Gram‐negative bacteria. Capsaicin extract from *Capsicum frutescens* demonstrates strong inhibition, especially against *Salmonella* and other pathogens, whereas ethanolic and methanolic extracts generally show higher efficacy than aqueous extracts. MIC and MBC values vary significantly, highlighting differences in potency based on fruit type, extraction method, and bacterial species.

## 4 Conclusions

The lycopene was extracted with a good yield from the selected fruits and vegetables by using an organic solvent. The absorbance peak of lycopene from different samples from 440 to 530 nm suggested the extraction of lycopene from the samples. The concentrations of lycopene present in the samples were in the following ascending order from lowest to highest concentration: (*C.a*.) < (*C.f*.) < (*C. p*.) < (*C. l*.) < (*S. l*.). The UV‐blocking property of *S. lycopersicum* was highest in all the conditions, whereas for *C. annuum* , it was lowest. The better UV‐blocking results of *S. lycopersicum* lycopene for *E. coli* suggest its application in cosmetics, especially sunburn cream. Such lycopene can be used in the cosmetics industry as a UV protectant. Further research and analysis are required to determine the effectiveness of lycopene, specifically in blocking exposure to UV rays when used in topical applications. Special attention will be significant in skincare and sunscreen formulations to ensure efficacy.

## Author Contributions

**Anil Kumar:** conceptualization (equal), methodology (equal), project administration (equal), supervision (equal), writing – original draft (lead), writing – review and editing (lead). **Niharika Bhawsar:** conceptualization (equal), investigation (equal), software (lead), visualization (lead), writing – review and editing (equal). **Savita Manekar:** conceptualization (equal), data curation (equal), investigation (supporting), software (equal), writing – review and editing (lead). **Bharat Pendram:** conceptualization (equal), investigation (equal), resources (equal), software (lead), validation (lead), writing – review and editing (supporting). **Pritibala Pal:** formal analysis (lead), investigation (lead), supervision (lead), validation (equal), writing – review and editing (equal). **Daoud Ali:** data curation (lead), funding acquisition (lead), resources (lead), software (lead), writing – review and editing (equal). **Saud Alarifi:** funding acquisition (lead), software (equal), validation (equal), writing – review and editing (supporting). **Shivraj Singh Wanale:** conceptualization (equal), project administration (equal), resources (lead), writing – review and editing (supporting). **Suchi Singh:** conceptualization (equal), formal analysis (equal), investigation (lead), visualization (equal), writing – review and editing (supporting). **Shailaja Katare:** data curation (lead), formal analysis (lead), investigation (equal), writing – review and editing (equal). **G. M. Srivastava:** conceptualization (equal), data curation (equal), investigation (supporting), methodology (lead), resources (equal), validation (supporting), visualization (equal), writing – review and editing (equal). **Parwiz Niazi:** funding acquisition (equal), investigation (equal), resources (equal), writing – review and editing (supporting). **Sakshi Pareek:** resources (supporting), software (equal), validation (equal), writing – review and editing (supporting). **Virendra Kumar Yadav:** formal analysis (equal), validation (supporting), visualization (supporting), writing – review and editing (supporting).

## Conflicts of Interest

The authors declare no conflicts of interest.

## Data Availability

All the data are present within the manuscript only.
